# REAPing the benefits: development and use of a structured evaluation framework to codify learning resources for Global Health professionals

**DOI:** 10.1186/s12909-021-02805-6

**Published:** 2021-07-08

**Authors:** Meike Schleiff, Elizabeth Hahn, Caroline Dolive, Lillian James, Anant Mishra, Bhakti Hansoti

**Affiliations:** 1 Department of International Health, Johns Hopkins Bloomberg School of Public, 615 N. Wolfe St., Baltimore, MD 21205 USA; 2 Sustaining Technical and Analytical Resources (STAR) project, Public Health Institute, Washington, DC, United States; 3 Department of Emergency Medicine, Johns Hopkins School of Medicine, Baltimore, MD USA

**Keywords:** Assessment, Competency, eLearning, Global Health, Leadership

## Abstract

**Background:**

The learning opportunities for global health professionals have expanded rapidly in recent years. The diverse array of learners and wide range in course quality underscore the need for an improved course vetting process to better match learners with appropriate learning opportunities.

**Methods:**

We developed a framework to assess overall course quality by determining performance across four defined domains Relevance, Engagement, Access, and Pedagogy (REAP). We applied this framework across a learning catalogue developed for participants enrolled in the Sustaining Technical and Analytic Resources (STAR) project, a global health leadership training program.

**Results:**

The STAR learning activities database included a total of 382 courses, workshops, and web-based resources which fulfilled 531 competencies across three levels: core, content, and skill. Relevance: The majority of activities were at an understanding or practicing level across all competency domains (486/531, 91.5%). Engagement: Many activities lacked any peer engagement (202/531, 38.0%) and had limited to no faculty engagement (260/531, 49.0%). Access: The plurality of courses across competencies were offered on demand (227/531, 42.7%) and were highly flexible in pace (240/531, 45.2%). Pedagogy: Of the activities that included an assessment, most matched activity learning objectives (217/531, 40.9%).

**Conclusions:**

Through applying REAP to the STAR project learning catalogue, we found many online activities lacked meaningful engagement with faculty and peers. Further development of structured online activities providing learners with flexibility in access, a range of levels of advancement for content, and opportunities to engage and apply learning are needed for the field of global health.

**Supplementary Information:**

The online version contains supplementary material available at 10.1186/s12909-021-02805-6.

## Background

A commitment to promoting societal health is perhaps one of the few unifying features of the global health and public health workforces [1, 2]. Those who work in the field approach their workplace challenges from different perspectives: as generalists and specialists; as academicians and field-based practitioners; as citizens of low- and middle-income countries (LMICs) and high-income countries; as advocates and clinicians [2–6]. Professionals at all stages of their career can benefit from opportunities to further develop and expand their skills and knowledge. The challenge is finding the right opportunities that best meet both their goals and professional needs. Moreover, given the plethora of learning options available, it is unclear how one should evaluate opportunities for suitability (i.e., appropriate content) versus desired format (e.g. in-person versus online, or a hybrid of both) and engagement level (e.g. interactive workshops versus asynchronous courses) [7, 8].

Recent years have seen an exponential rise in the availability of massive open online courses (MOOCs) and a rapid expansion in content variety and the number of institutions offering global health educational opportunities. Making online courses relevant, impactful, and desirable to learners has required drawing insights from both learning theory and learner demand [9, 10]. However, despite improvements, increased attention is still needed to appropriately design curricula that meet the skills and values sought by the diverse array of learners while still being actively engaging [10, 11]. The differing values of global health learners, combined with the continually expanding array of online resources, underscore the need for a systematic approach to catalogue the myriad learning opportunities available for global health professionals, many of whom are busy and have “numerous responsibilities that compete for their time” [12].

In recent years, as learning theories have been expanded to incorporate online education, a variety of course instruction rubrics have emerged as tools to help guide the development of new courses and better evaluate existing ones. Most of these rubrics have standards or indicators covering a general course overview and information, course design, learning objectives, accessibility, learner support, interaction and engagement, assessment, and technology (see Annex 1 for a summary of the topics covered by several commonly used rubrics) [13–16]. These rubrics have similarities as well as some unique features such as the degree they focus on communication between students and faculty and how outcomes are defined (e.g. baselines, effective, and exemplary results); most were designed for specific kinds of online learning activities and/or target audiences. We did not find a multi-purpose and vetted strategy that could be applied to the wide range of courses that global health professionals seek to build their skills. Thus, the Quality Matters (QM) rubrics, which have been tested and are widely utilized for online courses, outline eight general standards each with specific indicators and were utilized as a foundation for our tool [13].

The Sustaining Technical and Analytic Resource (STAR) project is a global health training program for both junior and experienced global health professionals funded by the United States Agency for International Development (USAID). The STAR curriculum and the cohorts are described in greater detail elsewhere [17]. These participants vary widely in their professional foci and backgrounds, with many focused on infectious disease programs (specifically tuberculosis or HIV/AIDS) and across a variety of technical areas (for example monitoring, evaluation, and learning (MEL) versus supply chain management). Participants provide technical support across a breadth of global health programs and are also provided with protected time and resources for leadership training and professional development. In order to facilitate identifying appropriate learning opportunities to match the needs of each unique participant, the STAR project built a database to catalogue hundreds of learning activities for participants across a diverse array of topic areas. We soon realized that the process of identifying, vetting, and assigning appropriate learning activities for a diverse pool of professionals was a more general obstacle for the field of global health to overcome.

This paper describes our experience developing and applying a framework to evaluate quality indicators for use by the STAR project. We document the initial process of sourcing and reviewing learning opportunities to develop our database of vetted learning activities and some key findings that can inform how other global health educational programs review existing curricula for potential use by their learners.

## Methods

### Background – STAR learning approach

**Fig. 1:**
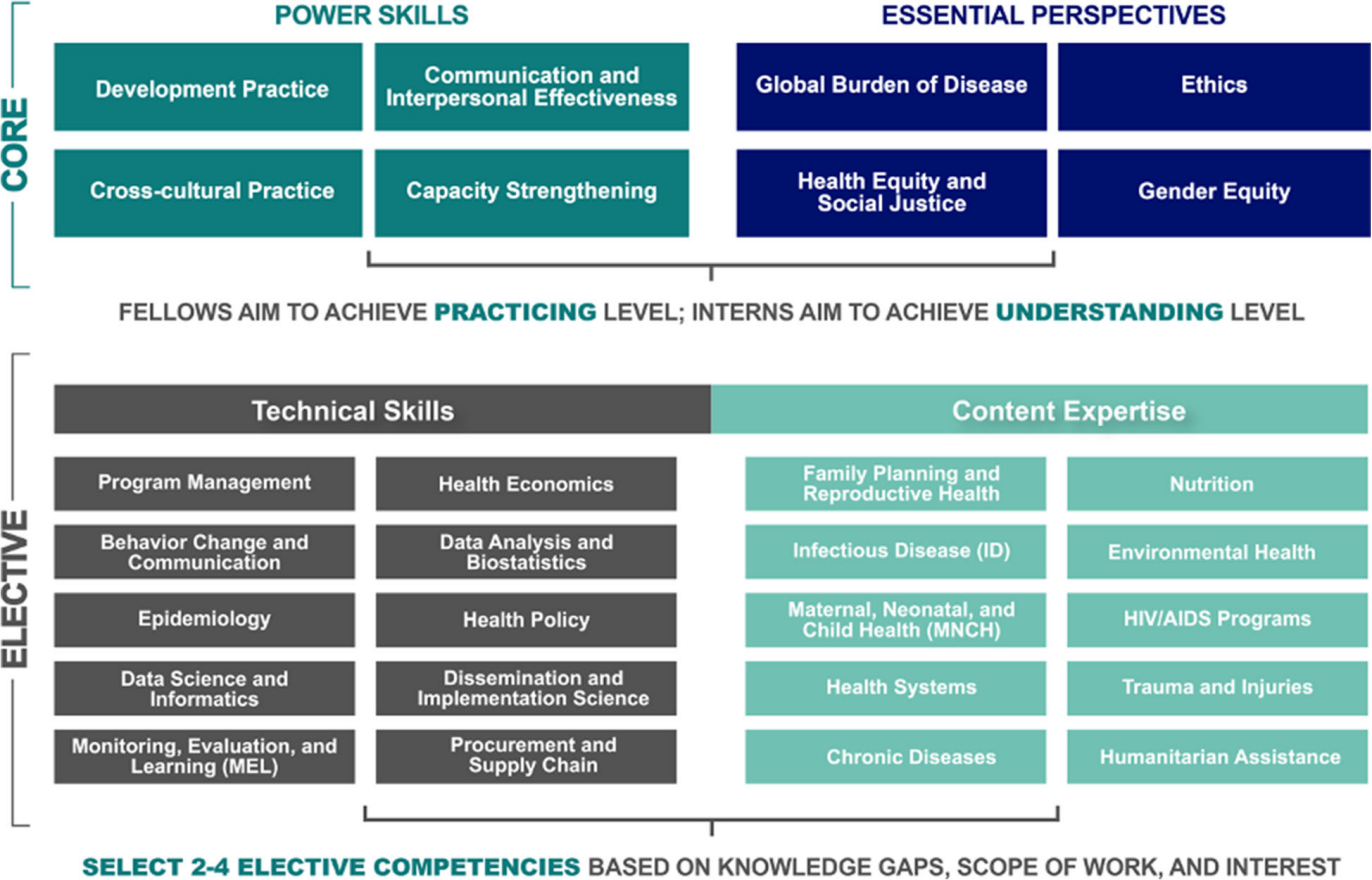
STAR Global Health Competencies Framework

STAR participants are provided with an individualized learning plan (ILP) that focuses on their individual development goals [17]. In order to guide the baseline competency assessments, development of ILPs, and the organization of our database, a competency framework was developed for STAR (see Fig. 1) [17]. This framework included “core” competencies that all participants were expected to demonstrate a minimum level of expertise in by the end of the fellowship as well as elective skill and content competencies. Within each competency domain, a set of five milestones were defined which represented demonstrable knowledge or skills ranging from a basic level of inquiry to advanced mastery.

### Development of the REAP tool

**Fig. 2:**
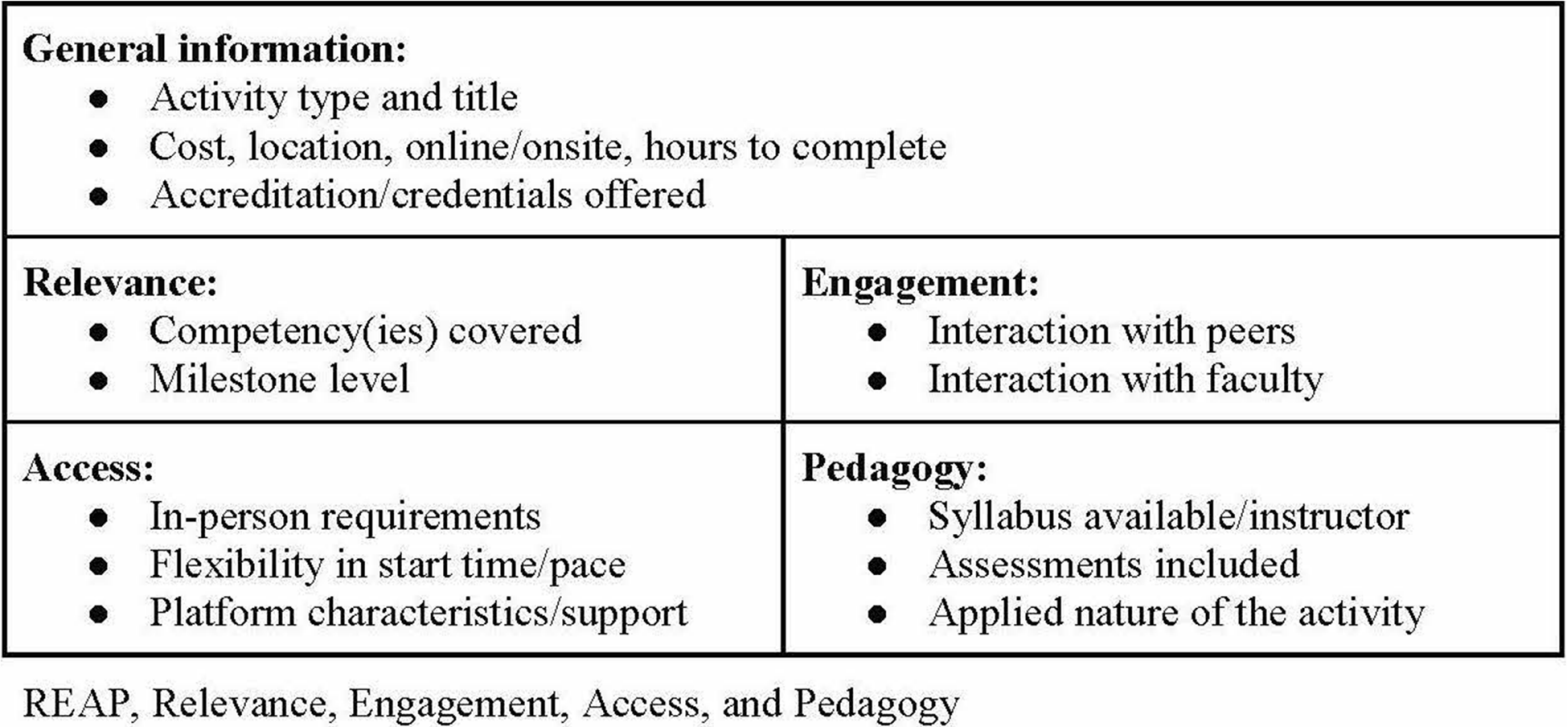
Key Variables Captured Within Each REAP Domain

We conducted an online review using Google, Google Scholar, PubMed, and the Education Resources Information Center (ERIC) for tools developed and implemented to assess course quality, particularly for online courses. Although STAR participants may also complete in-person courses, the rubrics we chose to look at primarily addressed online and blended courses as these were most likely to be the best fit for the majority of STAR participants and were where we anticipated finding the most variety in terms of the quality of these courses. Our goal was to design a framework that would first be broadly applicable to learning activities that the program was utilizing and developing, and second would provide us with a systematic approach to codifying the activities in our database to meet the needs and preferences of learners. Based on our review of the literature, and particularly the thoroughly tested and widely applied Quality Matters rubrics as a foundation, [13] the instructional design leads at the STAR project developed a tailored and systematic framework for codifying and vetting learning activities for STAR: The Relevance, Engagement, Access, and Pedagogy (REAP) tool (Fig. 2). The Relevance domain of REAP focuses on how well the content covered in a course or other activity aligns with STAR’s competency framework and milestone levels [17]. The Engagement domain focuses on the extent to which learners have opportunities to engage with each other and with course faculty. Within the Access domain, we capture key variables related to the format (online or onsite), pace, and flexibility of the learning activity. Finally, the Pedagogy domain captures elements of the course credibility and assessment approaches.

Given the wide range of backgrounds, levels of experience, and work contexts of global health and public health professionals, no single combination of course characteristics (e.g. online, self-paced, and with limited engagement with peers) will universally be the most desirable or appropriate for all participants. The most useful approach, therefore, was to establish a standard qualitatively oriented review process that could determine whether a course would meet the needs of a specific participant. Particularly for STAR’s diverse range of learners, we aimed not to determine definitively whether a course was “good” or “bad”, but rather to examine the fit of a particular activity with individual learner preferences and needs across a set of variables. To ensure consistency and defensibility of the reviews of each activity, we undertook an intensive and iterative approach entailing regular meetings to discuss database entries and included notes sections to provide explanations and document decisions during the data entry process in order to: 1) build shared understanding among all reviewers and 2) to provide detailed notes to ensure transparency and thick descriptions [18–20] for the decisions and any caveats related to each entry in the database. We also included a summary measure under each category of how good of a fit a particular learning activity was for STAR participants (for example, how well the Relevance variables for a particular activity aligned with the needs of STAR participants).

### Implementation of the REAP tool

We utilized the REAP tool to vet learning activities, including courses, workshops, and other activities, as they were entered into our learning activities database. Activities added to the database were identified through 1) an initial search for activities offered by STAR project partners and particularly focused on STAR core competencies [17] and 2) the specific areas of work and learning needs of STAR participants as they were onboarded. Each learning activity was reviewed by at least one STAR staff member and as much information as possible related to each REAP domain was added. The learning activity database is searchable by keyword as well as by select REAP indicators to locate opportunities that are the best fit for particular participants. Participant evaluations of learning activities are also accessible in the database so that participant satisfaction can be incorporated in the overall activity assessment.

Based on our experience using the tool, we made revisions to improve clarity and efficiency of the tool as we went along. These revisions did not change the content of the rubric itself and aimed to improve usability. Two key changes were the addition of summary measures for each category in order to provide an overall assessment of the fit of an activity related to a particular category (e.g. Relevance) for the majority of STAR participants. Secondly, we streamlined the number of open text response variables to make the tool faster to use and to better standardize our data.

### Contents of the REAP-vetted learning activities database

The learning database is live and was designed for STAR staff utilization in April 2019 and is added to continually. It contains a combination of workshops, courses (online and in-person), and web-based resources such as websites and grey literature (materials that are not controlled by commercial publishers) reports and manuals. Of note, the contents of the database reflect the learning needs of STAR participants and are not meant to be representative of the overall learning opportunities for global health and public health professionals.

### Data analysis

Data for this paper were pulled from the active STAR database on June 16, 2020. Data from the database were analyzed using Stata v.15 (StataCorp LLC, College Station, TX, USA). We chose to present findings disaggregated by STAR competency categories as a consistent way to analyze the REAP domains because of the centrality of these competencies to the design of STAR’s learning program and because we anticipated that some of the REAP variables would differ based on the kind of content that a learning activity focused on. A descriptive statistical approach was used to provide an overview of the learning activity database, characteristics of learning activities across each of the REAP domains, and the fit of each learning activity for STAR.

### Statement of IRB approval

Ethical approval was sought and received from the institutional review boards of the Public Health Institute (IRB #I19–022) and the Johns Hopkins Bloomberg School of Public Health (IRB00011259). Written consent was sought from all participants.

## Results

A total of 382 activities were evaluated by the REAP tool of which there are 40 workshops (10.5%), 280 Courses (73.3%) and 62 web-based resources (22.1%). Workshops included summer intensive workshops on qualitative research to management problem-solving sessions. Courses included both academic courses, those offered by USAID and partners, and trainings for private companies on a range of topics from gender equity to communications skills to languages. Web-based resources included self-paced training modules on tuberculosis from the Centers for Disease Control, a range of infectious disease resources including webinars and resource pages, and toolkits on scientific writing and data analysis. The full dataset that was utilized for our analysis of the database can be accessed in Additional file 1.

Table 1 further describes the characteristics of the included learning activities. The majority of learning activities were offered online (60% of workshops, 71.4% of courses, and 100% of web-based resources) and the majority of activities (85.1%) fulfilled 1–2 competencies. Most learning activities were based in the Pan American Health Organization (PAHO) region (84.8%) or European Regional Office (EURO) (9.2%) region, though some courses were also based in the African Regional Office (AFRO) countries (3.4%). Cost varied widely, with a large set of free courses (47.1%), but also a substantial set of courses costing over $1000 (22.5%).

**Table 1:** Characteristics of STAR learning database activities

	Workshop (*n* = 40)	Courses (*n* = 280)	Web-based Resources (*n* = 62)	Total (*N* = 382)
*Course Location*				
Online	24 (60.0%)	200 (71.4%)	62 (100.0%)	286 (74.9%)
In-person	9 (22.5%)	71 (25.4%)	0 (0.0%)	80 (20.9%)
Either	6 (15.0%)	5 (1.8%)	0 (0.0%)	11 (2.9%)
Unknown	1 (2.5%)	4 (1.4%)	0 (0.0%)	5 (1.3%)
*Fulfills Competency Categories*				
Core	29 (72.5%)	151 (53.9%)	15 (24.2%)	195 (51.0%)
Skill	19 (47.5%)	188 (67.1%)	34 (54.8%)	241 (63.1%)
Content	5 (12.5%)	69 (24.6%)	21 (33.9%)	95 (24.9%)
*Number of Competencies Fulfilled*				
0	0 (0.0%)	1 (0.4%)	1 (1.6%)	2 (0.5%)
1	12 (32.5%)	145 (51.8%)	47 (75.8%)	205 (53.7%)
2	20 (50.0%)	87 (31.1%)	13 (21.0%)	120 (31.4%)
3	4 (10.0%)	22 (7.9%)	0 (0.0%)	26 (6.8%)
4	1 (2.5%)	16 (5.7%)	1 (1.6%)	18 (4.7%)
5	1 (2.5%)	6 (2.1%)	0 (0.0%)	7 (1.8%)
6	1 (2.5%)	3 (1.1%)	0 (0.0%)	4 (1.1%)
*WHO Region*				
AFRO	0 (0.0%)	13 (4.6%)	0 (0.0%)	13 (3.4%)
EURO	2 (5.0%)	24 (8.6%)	9 (14.5%)	35 (9.2%)
PAHO	38 (95.0%)	238 (85.0%)	48 (77.4%)	324 (84.8%)
SEARO	0 (0.0%)	3 (1.1%)	1 (1.6%)	4 (1.1%)
WPRO	0 (0.0%)	2 (0.7%)	0 (0.0%)	2 (0.5%)
Unknown	0 (0.0%)	0 (0.0%)	4 (6.5%)	4 (1.1%)
*Cost*				
$0 (Free)	6 (15.0%)	117 (41.8%)	57 (91.9%)	180 (47.1%)
$1–$99	0 (0.0%)	46 (16.4%)	0 (0.0%)	46 (12.0%)
$100–$499	4 (10.0%)	32 (11.4%)	1 (1.6%)	37 (9.7%)
$500–$999	8 (20.0%)	12 (4.3%)	0 (0.0%)	20 (5.2%)
≥ $1000	21 (52.5%)	65 (23.2%)	0 (0.0%)	86 (22.5%)
Unknown	1 (2.5%)	8 (2.9%)	4 (6.45%)	13 (3.4%)
*Activity Hours*				
< 20	29 (72.5%)	158 (56.4%)	31 (50.0%)	218 (57.1%)
20–40	4 (10.0%)	47 (16.8%)	11 (17.7%)	62 (16.2%)
≥ 40	7 (17.5%)	75 (26.8%)	20 (32.3%)	102 (26.7%)
*Top 5 Institutions*				
Johns Hopkins University	2 (5.0%)	30 (10.7%)	3 (4.8%)	35 (9.2%)
American Management Assoc.	21 (52.5%)	13 (4.6%)	0 (0.0%)	34 (8.9%)
Coursera	0 (0.0%)	29 (10.4%)	0 (0.0%)	29 (7.6%)
USAID University	0 (0.0%)	24 (8.6%)	0 (0.0%)	24 6.3%)
World Health Organization	3 (7.5%)	10 (3.6%)	3 (4.8%)	16 (4.2%)

Cells highlighted blue signify the highest proportion

*STAR* Sustaining Technical and Analytical Resources

Each of the learning activities were further assessed across the four domains of the REAP tool. Results are displayed in Tables 2, 3, 4 and 5 with key variables for each domain broken down by the category of competency (core, content, or skill-based competency). Some activities were often able to fill multiple competencies and thus may be featured multiple times.

**Table 2:** REAP Relevance components by STAR competency category

	Core Competencies (*n* = 195)	Content Competencies (*n* = 95)	Skill Competencies (*n* = 241)	Total
*Milestones*				
Inquiring	4 (2.0%)	3 (3.2%)	13 (5.4%)	20
Understanding	74 (38.0%)	32 (33.7%)	89 (36.9%)	195
Practicing	106 (54.4%)	53 (55.8%)	132 (54.8%)	291
Leading	10 (5.1%)	7 (7.4%)	6 (2.5%)	23
Advancing	1 (0.5%)	0 (0.0%)	1 (0.4%)	2

*REAP* Relevance, Engagement, Access, and Pedagogy, *STAR* Sustaining Technical and Analytic Resources

**Table 3:** REAP Engagement components by STAR competency category

	Core Competencies (*n* = 195)	Content Competencies (*n* = 95)	Skill Competencies (*n* = 241)	Total
*Faculty Engagement*				
None	36 (18.5%)	38 (40.0%)	65 (27.0%)	139
Limited	41 (21.0%)	20 (21.0%)	60 (24.9%)	121
Average	27 (13.9%)	18 (19.0%)	53 (22.0%)	98
Variable	4 (2.1%)	2 (2.1%)	9 (3.7%)	15
High	83 (42.6%)	14 (14.7%)	50 (20.7%)	147
Unknown	4 (2.1%)	3 (3.2%)	4 (1.7%)	11
*Peer Engagement*				
None	60 (30.8%)	45 (47.4%)	97 (40.2%)	202
Limited	26 (13.3%)	12 (12.6%)	41 (17.0%)	79
Average	31 (15.9%)	20 (21.1%)	44 (18.3%)	95
Variable	9 (4.6%)	0 (0.0%)	8 (3.3%)	17
High	66 (33.8%)	14 (14.7%)	45 (18.7%)	125
Unknown	3 (1.5%)	4 (4.2%)	6 (2.5%)	13

Cells highlighted blue signify the highest proportion

*REAP* Relevance, Engagement, Access, and Pedagogy, *STAR* Sustaining Technical and Analytic Resources

**Table 4:** REAP Access components by STAR competency category

	Core Competencies (*n* = 195)	Content Competencies (*n* = 95)	Skill Competencies (*n* = 241)	Total
*In-person requirements*				
Full semester/term	13 (6.7%)	3 (3.2%)	7 (2.9%)	23
Workshop/training	63 (32.3%)	14 (14.7%)	53 (22.0%)	130
Optional	9 (4.6%)	2 (2.1%)	8 (3.3%)	19
Online only	110 (56.4%)	76 (80.0%)	173 (71.8%)	359
Unknown	0 (0.0%)	0 (0.0%)	0 (0.0%)	0
*Start flexibility*				
Infrequent start	47 (24.1%)	18 (19.9%)	56 (23.2%)	121
Frequent start	53 (27.2%)	2 (2.1%)	42 (17.4%)	97
Open enrollment	31 (15.9%)	9 (9.5%)	33 (13.7%)	73
On demand	58 (28.7%)	63 (66.3%)	106 (44.0%)	227
Unknown	6 (3.1%)	3 (3.2%)	4 (1.7%)	13
*Pace flexibility*				
Fixed pace	52 (26.7%)	19 (20.0%)	58 (24.1%)	129
Designated schedulewith own pace	44 (22.6%)	2 (2.1%)	32 (13.3%)	78
Flexible	25 (12.8%)	6 (6.3%)	38 (15.8%)	69
Highly flexible	68 (34.9%)	66 (69.5%)	106 (44.0%)	240
Unknown	6 (3.1%)	2 (2.1%)	7 (2.9%)	15

Cells highlighted blue signify the highest proportion

*REAP* Relevance, Engagement, Access, and Pedagogy, *STAR* Sustaining Technical and Analytic Resources

**Table 5:** Pedagogy components of REAP by STAR competency category

	Core Competencies (*n* = 195)	Content Competencies (*n* = 95)	Skill Competencies (*n* = 241)	Total
Syllabus				
Provided	93 (47.7%)	37 (38.9%)	133 (55.2%)	263
Not provided	81 (41.5%)	38 (40.0%)	80 (33.2%)	199
Unknown	21 (10.8%)	20 (21.1%)	28 (11.6%)	69
Instructor Credibility				
Credible	164 (84.1%)	83 (87.4%)	203 (84.2%)	450
Unknown/Not credible	31 (15.9%)	12 (12.6%)	38 (15.8%)	81
Activity Application				
High	27 (13.9%)	6 (6.3%)	27 (11.2%)	60
Average	56 (28.7%)	17 (17.9%)	37 (15.3%)	110
Limited	56 (28.7%)	36 (37.9%)	106 (44.0%)	198
None	11 (5.6%)	14 (14.7%)	18 (7.5%)	43
Unknown	45 (23.1%)	22 (23.2%)	53 (22.0%)	120
Assessment Inclusion				
Aligned	88 (45.1%)	24 (25.3%)	105 (43.6%)	217
Misaligned	4 (2.1%)	0 (0.0%)	1 (0.4%)	5
Unexplained	18 (9.2%)	16 (16.8%)	25 (10.4%)	59
Not included	29 (14.9%)	24 (25.3%)	37 (15.3%)	90
Inclusion Unclear	56 (28.7%)	31 (32.6%)	73 (30.3%)	160

Cells highlighted blue signify the highest proportion

*REAP* Relevance, Engagement, Access, and Pedagogy, *STAR* Sustaining Technical and Analytic Resources

### Relevance

The relevance findings, summarized in Table 2, describe how the learning activities in the database were distributed across the three categories of STAR competencies (core, content, and skill) and by milestone level. Most courses across all competencies were either at the understanding (195/531, 36.7%) or practicing level (291/531, 54.8%). Very few courses were at the inquiring (20/531, 3.8%) or leading level (2/531, 0.4%). Many activities were identified as highly relevant if they addressed core competency domains and were geared towards understanding and practicing levels, as these courses were relevant a substantial proportion of STAR participants to meet minimum requirements or the STAR experience and/or to enhance these core skill areas. For example, courses on gender and health equity as well as language learning opportunities were identified as relevant for the STAR audience.

### Engagement

Engagement was a REAP domain that STAR participants prioritized, with general preferences for higher levels of engagement with faculty and peers, which are presented in Table 3. Among the core competency-related activities, there was a high level of faculty engagement (83/195, 42.6%), but about equally as many had either no direct faculty engagement (36/195, 18.5%) or limited engagement (41/195, 21.0%). Faculty engagement levels also varied for the content and skill competencies, though skill competencies had a number of activities with average (53/241, 22.0%) and high levels of engagement (50/241, 20.7%). High engagement activities tended to be workshops and consultative meetings and groups with a focus on peer dialogue and problem solving, such as a Technical Consultation on Expanding Contraceptive Method Choice that was included in our database. Low engagement courses included self-paced online courses such as a Coursera course on supply chain management.

For peer engagement, many activities across all competency categories had no engagement (core: 60/195, 30.8%; content: 45/95, 47.4%; skill: 97/241, 40.2%). For activities covering core competencies, the largest proportion of activities were characterized as having high levels of engagement (66/195, 33.8%). For content competency-related activities, the second largest set of courses had an average amount of peer engagement (20/95, 21.1%). Finally, the skill competency activities were fairly equally distributed across limited (41/241, 17.0%), average (44/241, 18.3%), and high (45/241, 18.7%) levels of engagement.

### Access

Access variables include the in-person requirements of an activity and the flexibility in start time and pace as presented in Table 4. The largest proportion of courses were offered online only across all categories of competencies (core: 110/195, 56.4%; content: 76/95, 80.0%; skill: 173/241, 71.8%). Core competency-related activities also had a large proportion (63/195, 32.3%) offered in workshop or short training formats, while the content and skill competency-related activities had a small, but still noteworthy, number of these workshop format activities (content: 14/95, 14.7%; skill 53/241, 22.0%). Many language offerings, such as immersion programs for foreign languages, require in person participation whereas any courses has online options available.

For core competency activities, the flexibility in start time varied and was spread fairly evenly across infrequent starts (once or twice a year) (47/195, 24.1%), frequent starts (53/195, 27.2%), and on demand courses (available whenever learners signed up) (58/195, 28.7%). Skill competencies followed a similar distribution, but with more courses (106/241, 44.0%) offered on demand. Content-related activities were more commonly available on demand (63/95, 66.3%).

Across competencies, the most common amount of flexibility in terms of the pace at which a participant could complete the course was “highly flexible” (core: 68/195, 34.9%; content: 66/95, 69.5%; skill: 106/241, 44.0%).

### Pedagogy

Table 5 includes a set of variables related to the pedagogy of each activity. For this variable, we evaluated the availability of a syllabus, the credibility of the instructor, the applied learning aspects, and use of assessment as a learning tool. Activities were split between which had and which did not have formal syllabi: core competency-related activities provided a syllabus (93/195, 47.7%) more often than not (81/195, 41.5%) and more skills activities also had a syllabus (133/241, 55.2%) than did not (80/241, 33.2%). The vast majority of instructors for all categories of activities were considered to be credible (core: 164/195, 84.1%; content: 83/95, 87.4%; skill: 203/241, 84.2%).

The amount of application of knowledge and concepts in an activity was also a priority for STAR, with many participants valuing higher levels of applicability to both real-world examples and their work. The amount of applicability found within activities varied, but for many activities, there was not enough information about this aspect available (core: 45/195, 23.1%; content: 22/95, 23.2%; skill: 53/241, 22.0%). An example of a highly applied activity was a course on graphic design and digital presentations, whereas others courses such as ones becoming a better teacher or core principles of communications received scores for lower or no direct application to participants’ work.

In terms of the inclusion and alignment of learner assessment within activities, both the core (88/195, 45.1%) and skill (105/241, 43.6%) competency-related activities had substantial proportions that included assessments that were highly aligned with activity learning objectives and topics. However, all categories of competencies had almost one third of activities for which the inclusion of any assessments was unknown (core: 56/195, 28.7%; content: 31/95, 32.6%; skill: 73/241, 30.3%).

## Discussion

The REAP tool presents a structured approach to codify learning activities in a systematic way. We were able to successfully apply the REAP tool to the STAR learning activities database and found it to be useful when identifying potential gaps and specific activities to meet learning needs of STAR participants. Our database, due to the needs of our participants, boosts a high volume of online learning resources. As such, we found that there are a large number of activities that had flexible pace and were easily accessible. This focus on online courses also meant that many of the activities were at an introductory level, syllabi were often not available, and a large number provide limited faculty or peer engagement opportunities.

The REAP tool adds to the available strategies designed to help educators and trainees sort through the plethora of available learning opportunities. As online education has expanded, educators have developed principles to guide the development of online learning. Effective online learning includes interactive and collaborative learning through synchronous discussions and reflections through asynchronous tools. Within these activities, instructors can create a safe and educational learning environment for learners by encouraging the development of critical thinking, monitoring discussion fora, and providing guidelines to ensure that course content and discussions are grounded in factual information [10]. In addition, online education needs to be both adaptable to the needs of diverse learners through varied formats to deliver content as well as flexible in order to allow learners to navigate content at their own pace [9]. While these criteria are not novel, our analysis showed that many of the activities available to our participants lacked an emphasis on meaningful engagement (which was identified as a high priority consideration by many STAR participants) and adopted a more passive approach to content delivery. Therefore, those activities were found to be less ideal for the particular audience of the majority of STAR participants.

A central consideration in course design and evaluation is understanding the components students’ value. For example, both young professional students as well as “nontraditional” (e.g. mid-career and executive-level professionals) students have been found to place similar importance on how a course is assessed, but nontraditional students place greater importance on how well a course is designed, especially with regards to course expectations and instructions for accessing resources and support [12]. Two past evaluations have found that services are needed to support learners based on their individual needs and that most students valued peer interaction and instructor feedback [21, 22]. Our experience using REAP to identify learning activities for participants led us to recognize that there is still a lack of online courses that take full advantage of pedagogical and technological resources available to engage learners, which needs to be addressed in order to meet the needs presented in the literature as well as priorities voiced by STAR participants.

While this paper provides a practical example of how a structured evaluation tool can be used to codify learning activities, it is not without limitations. Principally, the activities in the STAR database do not represent the plethora of global health resources available, but rather a subset of activities that were chosen for STAR participants based on their individual goals and work needs. As such, this paper is not intended to be a comprehensive evaluation of global health learning opportunities, but rather a case example presenting how an innovative structured evaluation approach to identifying appropriate learning opportunities for diverse learners can be implemented as part of a global health training program. An additional limitation is that, while we integrated an intensive qualitative approach to ensuring the rigor and consistency of the content in this database, additional quantitative validity checks were beyond the scope of this project and thus present an important opportunity for further research.

## Conclusion

This paper provides a description of our development and use of the REAP framework as well as the results of our initial experience utilizing it in the STAR project. While the REAP tool does not serve as a formal assessment tool to determine which courses are “better” or “worse” (and thus will be unable to provide an approach to ranking activities), as a project team we found the use of this tool to be a useful exercise to allow us rapidly identify the core characteristics of an activity in a systematic, standardized way and match these with participants who required particular content, interaction with faculty and peers, level of flexibility in delivery mode, and applicability to their job and career goals. Such tools will continue to become more valuable as the quantity and diversity of global health learning activities continues to expand. We hope that our experience developing and using REAP within the context of the STAR project can be a valuable example for other global health training programs to adapt and learn from.

### Supplementary Information

**Additional file 1.**

## Data Availability

The data utilized for this analysis are available as an Additional file 1 to this paper according to the United States Agency for International Development (USAID)‘s Public Access Plan.

## Annex 1

**Table 6:** Summary of key standards of commonly used course assessment rubrics

Rubric	Standard Categories
Specific Review Standards from the Quality Maters (QM) Higher Education Rubric, Sixth Edition	• Course Overview and Introduction • Learning Objectives (Competencies) • Assessment and Measurement • Instructional Materials • Learning Activities and Learner Interaction • Course Technology • Learner Support • Accessibility and Usability
California State University, Chico Rubric for Online Instruction	• Learner Support & Resources • Online Organization & Design • Instructional Design & Delivery • Assessment & Evaluation of Student Learning • Innovative Teaching with Technology • Faculty Use of Student Feedback
Illinois Online Network (ION) Quality Online Course Initiative (QOCI)	• Instructional Design • Communication, Interaction, and Collaboration • Student Evaluation & Assessment • Learner Support and Resources • Instructional Materials & Technologies • Accessibility • Course Evaluation
University of Minnesota College of Education and Human Development Digital Education and Innovation Team The Check: A Guide to Online Course Design	• Course Information • Course Design • Graphic Design • Student Support • Learning Objectives • Activities & Assignments • Assessment/Grading • Materials & Media • Accessibility & Universal Design • Course Communication • Learning Technologies • Course Evaluation

## Abbreviations

AFRO: African Regional Office
ERIC: Education Resources Information Center
EURO: European Regional Office
ILP: Individualized learning plan
LMIC: Low- and Middle-Income Countries
MEL: Monitoring, evaluation, and learning
MOOC: Massive Open Online Courses
PAHO: Pan American Health Organization
QM: Quality Matters
REAP: Relevance, Engagement, Access, and Pedagogy
STAR: Sustaining Technical and Analytic Resources
USAID: United States Agency for International Development
